# Supplementary material for: Transposon-activated POU5F1B promotes colorectal cancer growth and metastasis
Source: Nat Commun. 2022 Aug 20;13:4913. doi: 10.1038/s41467-022-32649-7 (PMC9392749; doi:10.1038/s41467-022-32649-7)
Supplement: Supplementary file 1 — Supplementary Information [file 41467_2022_32649_MOESM1_ESM.pdf]

Supplementary Figure 1

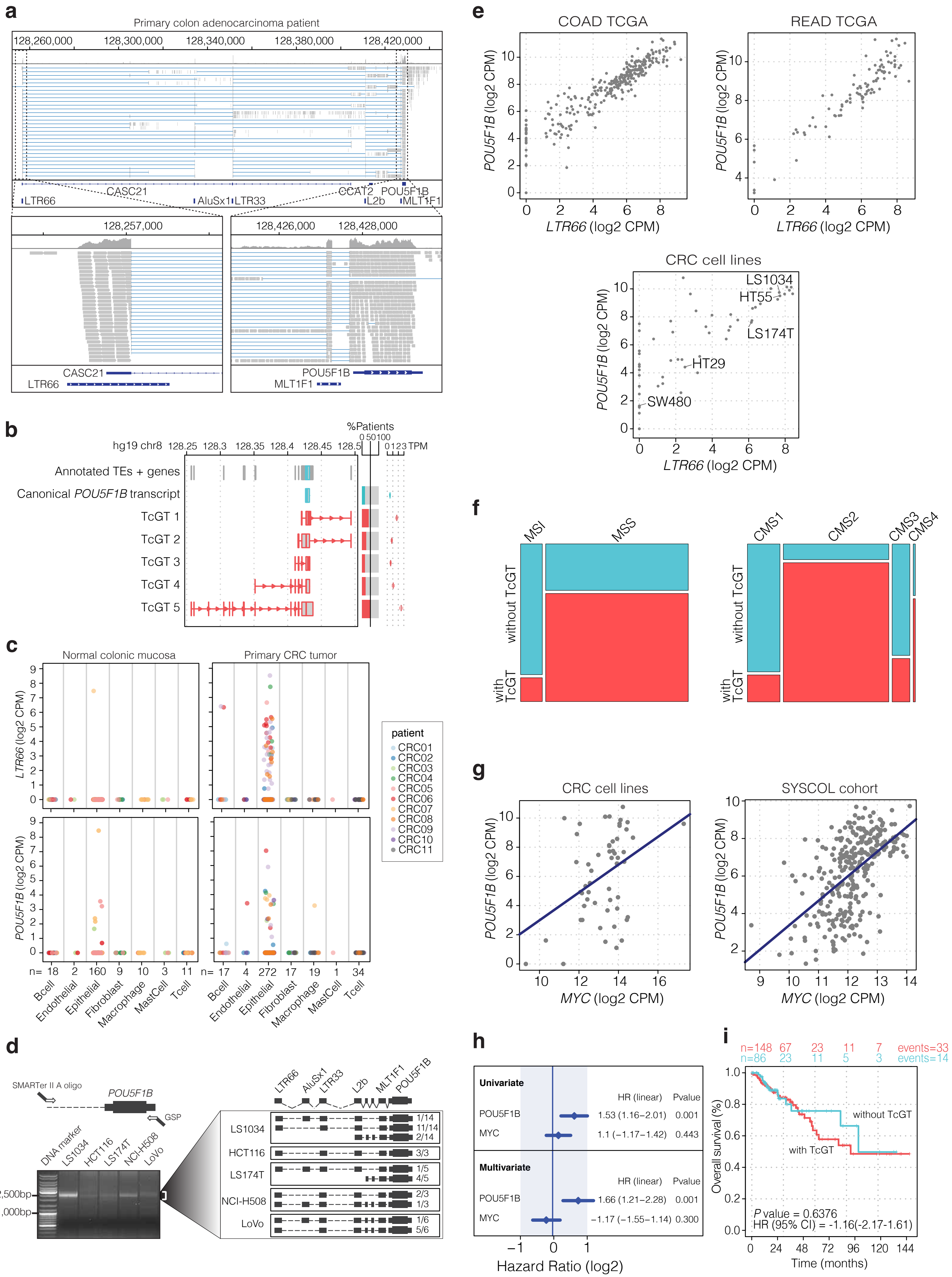

Supplementary Figure 1. POU5F1B transcripts in CRC. a, RNA-seq data are displayed in the Integrative Genomics Viewer (IGV). Human genome hg19 chr8 coordinates, coverage, read alignment, annotated genes and TEs are depicted, with grey bars representing sequencing reads and blue lines those spanning splice junctions. LTR66-POU5F1B transcripts from a primary colon adenocarcinoma patient from the SYSCOL cohort, with splicing from LTR66 into several other TE inserts (AluSx1, LTR33, L2b, MLT1F1) before reaching the POU5F1B-coding sequence. A detailed view of the TcGT 5' and 3' ends is provided. **b,** Assembly of all POU5F1B transcripts detected in the SYSCOL RNAseq data. Human genome hg19 chr8 coordinates with annotated TEs (grey bars) and POU5F1B gene (blue bar), with canonical transcript and 5 different TcGTs, the first two of which extend up to a TE located 3' of POU5F1B. Relative frequency (% of patients) and levels (TPM, transcripts per million) are indicated on the right. LTR66-POU5F1B (number 5) is the most frequent and highly expressed TcGT. Due to the potentially limited processivity of the reverse transcriptase, we cannot exclude that some of the shorter TcGTs were artificially 5' truncated. **c,** Single-cell RNA-seq data from 11 primary CRC tumors and matched normal samples, distributed in seven cell types[17], with number of cells in each category (n) indicated underneath. Reads mapped on LTR66 and POU5F1B are mainly detected in epithelial cells. **d,** Left, agarose gel analysis of 5'-RACE products after amplification with a gene-specific primer (GSP) and the SMARTer II A oligonucleotide from the SMARTer RACE technology. Most amplification products are about 2,500 bp in length, whereas the mono-exonic annotated POU5F1B transcript (~1,000 bp) was rarely amplified. Right, schematic structures and relative frequencies of ~2,500 bp-long mRNA species recovered from indicated CRC cell lines. An experiment representative of three is shown. **e,** Correlation plots of POU5F1B and LTR66 RNA expression in indicated tumors (COAD and READ, colon and rectum adenocarcinoma, respectively) from the TCGA dataset (two-sided Pearson estimate = 0.92 and = 0.92; P = 4.12e−120 and = 1.39e-40, respectively) and in 54 CRC cell lines from the EGAD00001000725 repository[18] (Pearson estimate = 0.8; P = 4.33e−13), pointing to the five cell lines used in the present study. The expression of TcGT was detected in LS1034, HT55 and LS174T, but not in HT29 and SW480. **f,** Mosaic plot representing the occurence of POU5F1B TcGTs in MSI/MSS (left) or CMS1-4 (right) tumors from SYSCOL cohort. **g,** Correlation plot of POU5F1B and MYC RNA expression in the 54 CRC cell lines from the EGAD00001000725[18] repository (Pearson estimate = 0.4, P = 0.0026) and the SYSCOL cohort (two-sided Pearson estimate = 0.56, P = 9.385e−26). **h,** Survival analysis with MYC and POU5F1B as independent predictors (univariate) of survival, or considering them under the presence of the other one as a covariate (multivariate). A Cox Proportional Hazards model was fitted to compute Hazard Ratios and Pvalues. **i,** Kaplan-Meier representation of overall survival in stages II-III TCGA patients according to presence (n = 148) or absence (n = 86) of POU5F1B TcGT. HR and CI were computed by fitting univariate Cox model.

Supplementary Figure 2

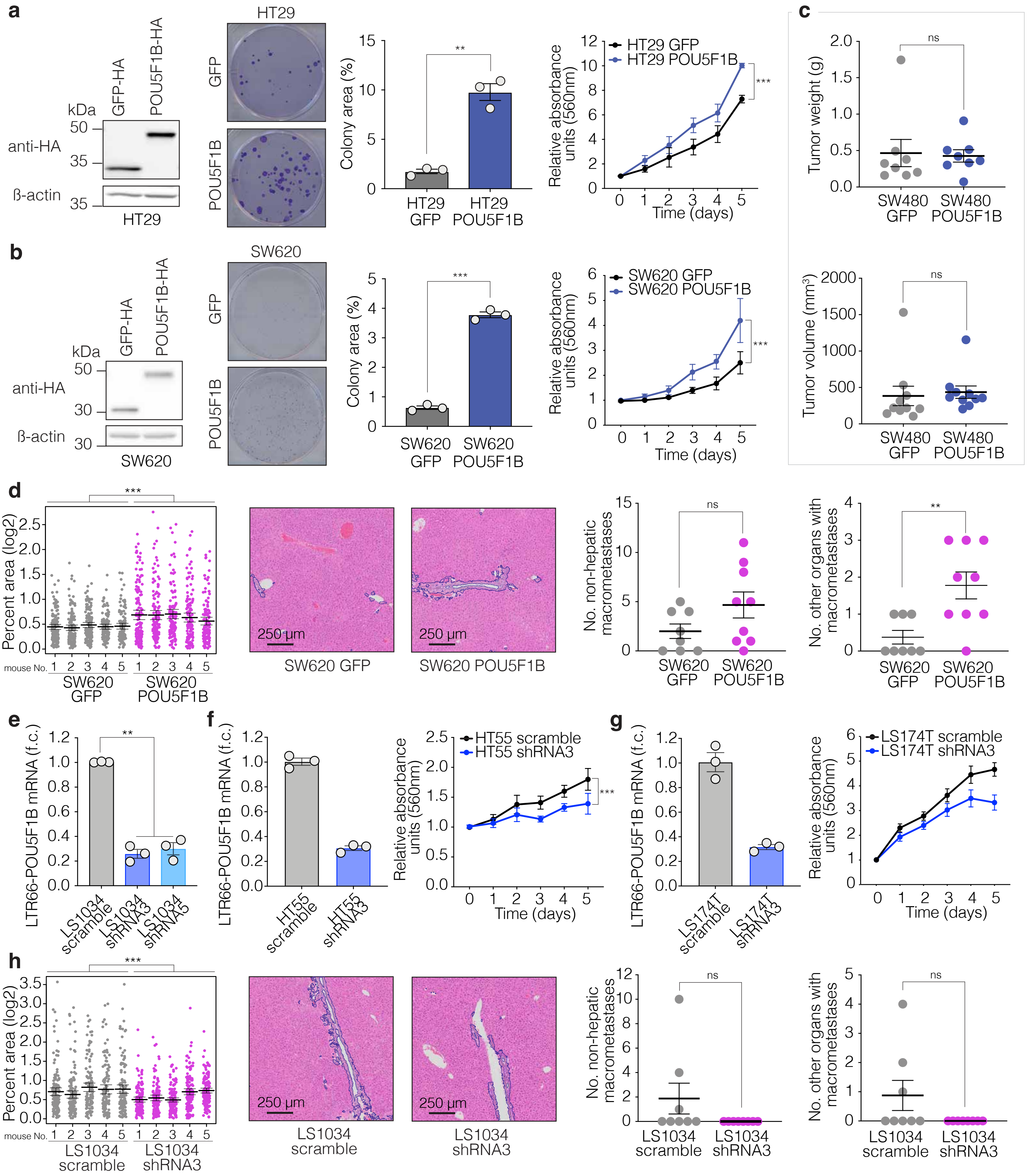

Supplementary Figure 2. POU5F1B enhances progression in CRC cell lines. a, Left, western blot of HA-tagged POU5F1B and GFP in transduced HT29 cells; middle, colony formation assay (CFA) (n = 3 independent experiments; P = 6.7e-03 by two-sided t-test); right, 3-(4,5-dimethylthiazol-2-yl)-2,5-diphenyltetrazolium bromide (MTT) assay (n = 3 independent experiments each with 12 replicates; P = 1.58e-07 by two-sided Wilcoxon test). **b,** Left, western blot of HA-tagged POU5F1B and GFP in transduced SW620 cells; middle, colony formation assay (CFA) (n = 3 independent experiments; P = 5.7e-05 by two-sided t-test test); right, MTT assay (n = 3 independent experiments each with 12 replicates; P = 3.05e-09 by two-sided Wilcoxon test). **c,** Tumor weight and volume at the implantation site (n = 10 animals per group, P = 0.25 and 0.98 by two-sided t-test). **d,** Percentage of liver area (log2) occupied by micrometastases, microscopy of H&E-stained slices, number of non-hepatic macrometastases, and additional organs affected by macrometastases following intrasplenic injection of POU5F1B- and GFP-transduced SW620 cells and splenectomy (n = 9 for POU5F1B-overexpressing, 8 for GFP control, P = 3.1e-17, P = 0.102, P = 0.005 respectively, by two-sided t-test). **e,** Quantitative PCR of LTR66-POU5F1B mRNA (n = 3 independent experiments; P = 3.86e-04 by two-sided t-test) of LS1034 cells transduced with two different inducible TcGT-targeting shRNAs or a control scramble sequence. **f, g,** Left, quantitative PCR (qPCR) of LTR66-POU5F1B mRNA of HT55 and LS174T cells transduced with one TcGT-targeting shRNA or a control scramble sequence; right, MTT assay (n = 3 independent experiments each with 6 replicates, P = 5.97e-04, by two-sided Wilcoxon test; n = 2 independent experiments each with 6 replicates, respectively). **h,** Percentage of liver area (log2) occupied by micrometastases, microscopy of H&E-stained slices, number of non-hepatic macrometastases, and additional organs affected by macrometastases following intrasplenic injection of LS1034 cells and splenectomy (n = 8 animals per group, P = 2.7e-8, P = 0.076, P = 0.076 respectively, by two-sided t-test). A blue line delimits the quantified area in representative microscopy of H&E-stained slices in **d** and **h**. Data in (a-h) are presented as mean values ± s.e.m., with single values as circles. *P < 0.05, **P < 0.01, ***P <0.001. Source data are provided as a Source Data file.

Supplementary Figure 3

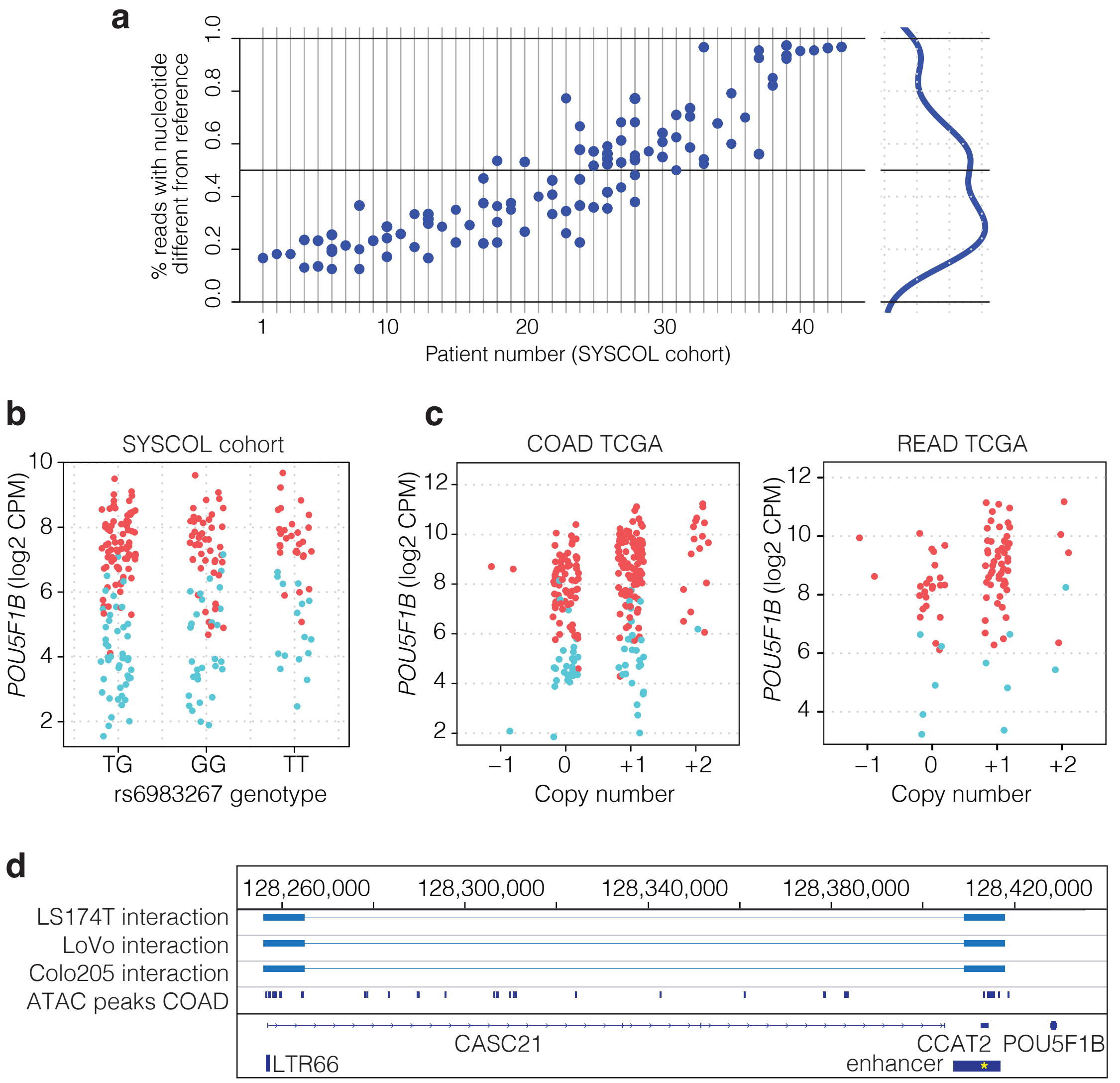

Supplementary Figure 3. Genomic features associated with LTR66-POU5F1B expression. a, Allelic repartition of POU5F1B expression in SYSCOL cohort patients. 43 patients presenting intraindividual SNPs in sequences for which there were more than 20 RNAseq reads are represented. **b,** Levels of POU5F1B expression as a function of the cancer-associated variant rs6983267 in the SYSCOL cohort (P = ns, by t-test). **c,** Levels of POU5F1B expression as a function of copy number variations in COAD and READ cohorts from the TCGA (-1 = hemizygous deletion; 0 = neutral / no change; 1 = gain; 2 = high-level amplification) (P = ns, by t-test). Samples in b-c are colored according presence (red dot) or absence (blue dot) of POU5F1B TcGTs. **d,** IGV screenshot of the LTR66-POU5F1B region including the Capture Hi-C interactions found in LS174T, LoVo, and Colo205 CRC cell lines[25]; and the ATACseq peaks from COAD TCGA[26]. Gene track and simplified TE and enhancer tracks are depicted. The rs6983267 locus is indicated by a yellow star.

Supplementary Figure 4

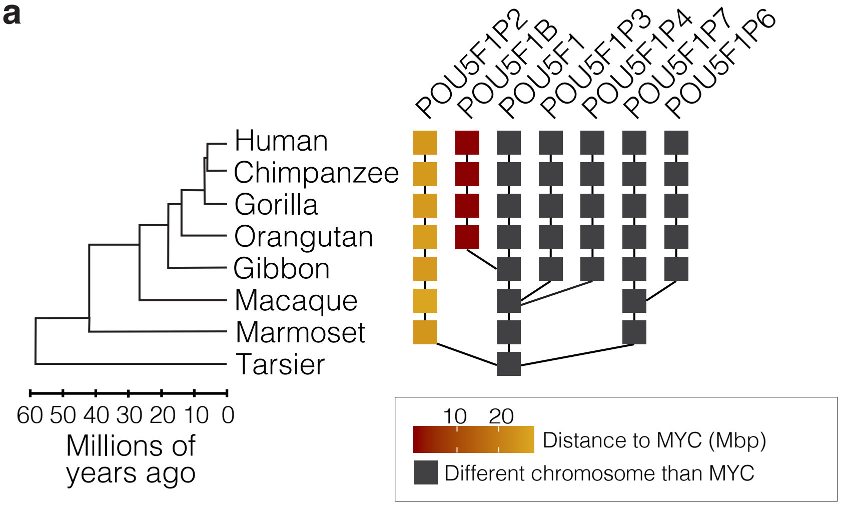

b

```
POU5F1B-359aa          MAGHLASDFAFSPPPGGGGDGPWGAEPGWVDPLTWLSFQGPPGGPGIGPGVGPGSEVWGI
POU5F1 -360aa          MAGHLASDFAFSPPPGGGGDGPGGPEPGWVDPRTWLSFQGPPGGPGIGPGVGPGSEVWGI
                       ********************* * ******* ****************************

POU5F1B-359aa          PPCPPPYELCGGMAYCGPQVGVGLVPQGGLETSQPESEAGVGVESNSNGASPEPCTVPPG
POU5F1 -360aa          PPCPPPYEFCGGMAYCGPQVGVGLVPQGGLETSQPEGEAGVGVESNSDGASPEPCTVTPG
                       ********:***************************.**********:********* **

POU5F1B-359aa          AVKLEKEKLEQNPEKSQDIKALQKELEQFAKLLKQKRITLGYTQADVGLILGVLFGKVFS
POU5F1 -360aa          AVKLEKEKLEQNPEESQDIKALQKELEQFAKLLKQKRITLGYTQADVGLTLGVLFGKVFS
                       **************:********************************** **********

POU5F1B-359aa          QKTICRFEALQLSFKNMCKLRPLLQKWVEEADNNENLQEICKAETLMQARKRKRTSIENR
POU5F1 -360aa          QTTICRFEALQLSFKNMCKLRPLLQKWVEEADNNENLQEICKAETLVQARKRKRTSIENR
                       *.********************************************:*************

POU5F1B-359aa          VRGNLENLFLQCPKPTL-QISHIAQQLGLEKDVVRVWFCNRRQKGKRSSSDYAQREDFEA
POU5F1 -360aa          VRGNLENLFLQCPKPTLQQISHIAQQLGLEKDVVRVWFCNRRQKGKRSSSDYAQREDFEA
                       ***************** ******************************************

POU5F1B-359aa          AGSPFSGGPVSFPPAPGPHFGTPGYGSPHFTALYSSVPFPEGEVFPPVSVITLGSPMHSN
POU5F1 -360aa          AGSPFSGGPVSFPLAPGPHFGTPGYGSPHFTALYSSVPFPEGEAFPPVSVTTLGSPMHSN
                       ************* *****************************.****** *********
```

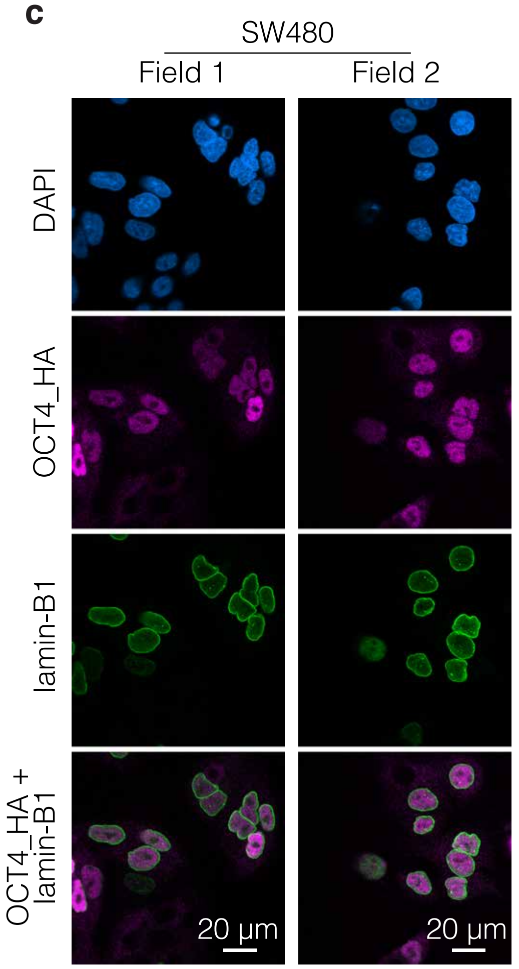

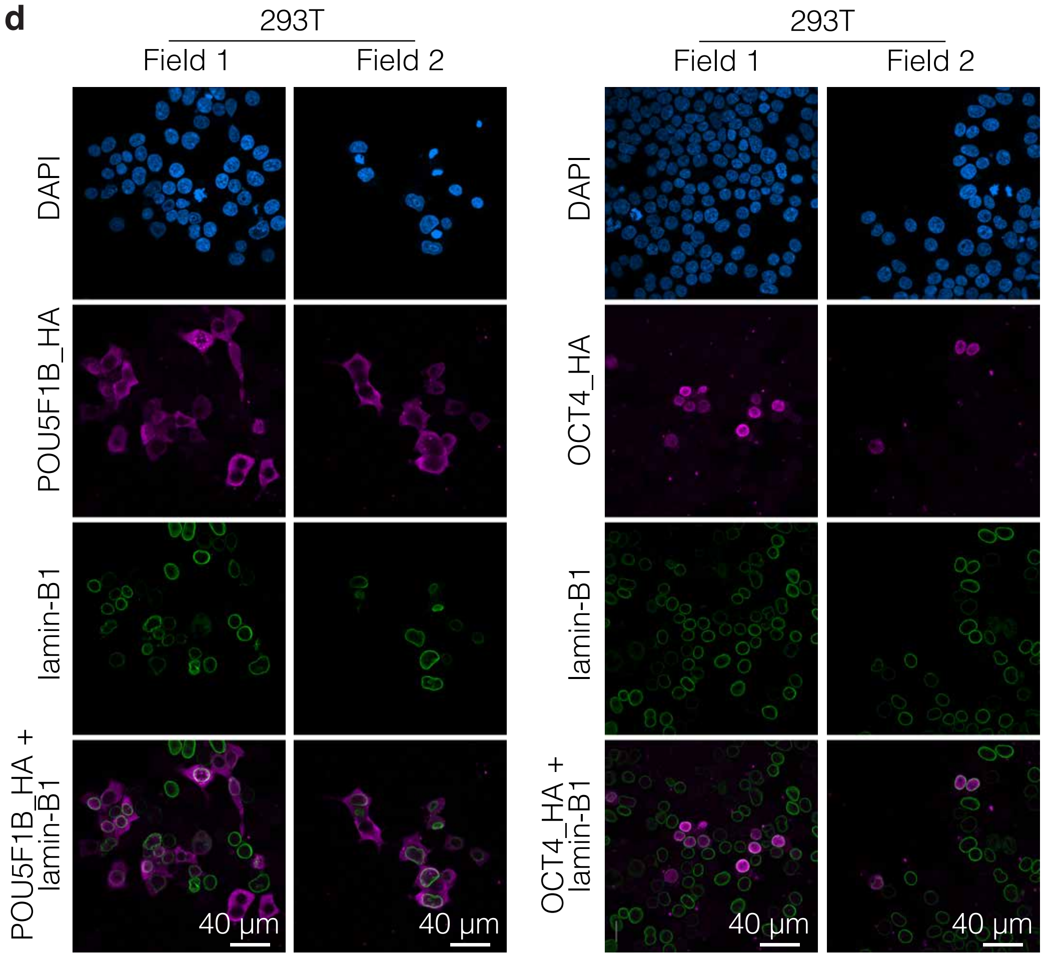

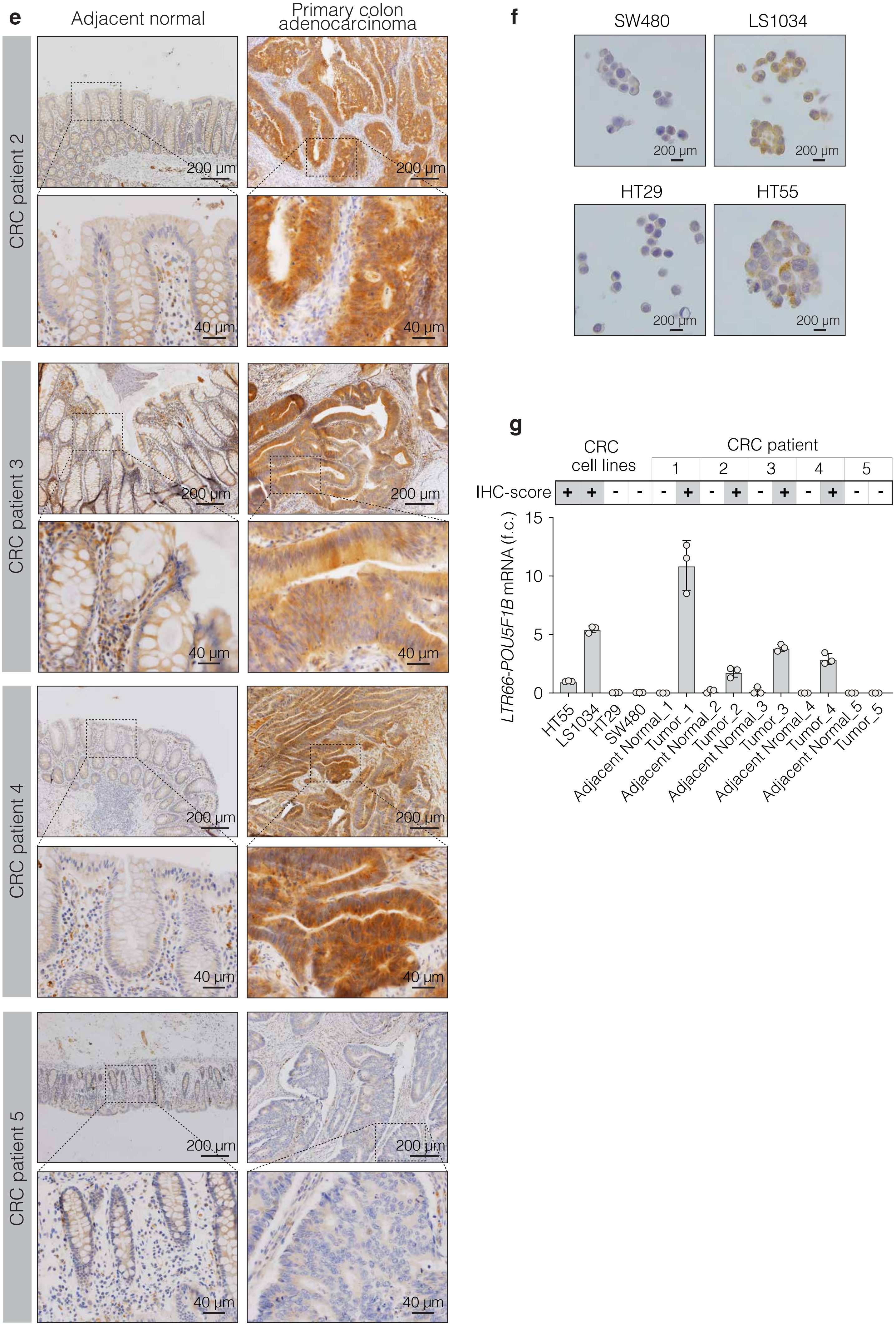
e
Adjacent normal
Primary colon adenocarcinoma
CRC patient 2
CRC patient 3
CRC patient 4
CRC patient 5
200 µm
40 µm
f
SW480
LS1034
HT29
HT55
200 µm
g
CRC cell lines
CRC patient
1
2
3
4
5
IHC-score
+ + - - - + - + - + - + - -
LTR66-POU5F1B mRNA (f.c.)
15
10
5
0
HT55
LS1034
HT29
SW480
Adjacent Normal_1
Tumor_1
Adjacent Normal_2
Tumor_2
Adjacent Normal_3
Tumor_3
Adjacent Nromal_4
Tumor_4
Adjacent Normal_5
Tumor_5

Supplementary Figure 4. Salient differences between POU5F1B and OCT4. a, Evolutionary tree based on syntenic sequences comparison of POU5F1B and its six closest paralogs (>80% homology with human POU5F1B) between human and tarsier, marmoset, macaque, gibbon, orangutan, gorilla and chimpanzee. Chromosomal distance to MYC (in Mbp) is depicted by a color gradient from yellow (far away) to red (close). Dark-grey indicates sequences in another chromosome than MYC. **b,** Alignment of POU5F1B (359 aa) and POU5F1 (360 aa) protein sequences. 15 amino acid (aa) differences are highlighted in pink. **c,** Representative immunofluorescence imaged by confocal microscopy of OCT4-HA-overexpressing SW480 cells, with DAPI (blue) and antibodies against HA (pink) and the nuclear membrane marker lamin-B1 (green). Two fields out of 8 are shown. **d,** Representative immunofluorescence-confocal microscopy of POU5F1B-HA (left) and OCT4-HA (right) overexpressing 293T cells, with DAPI in blue, HA in pink and the nuclear membrane marker lamin-B1 in green. Two fields out of eight are shown. **e,** Immunohistochemistry for endogenous POU5F1B in adjacent normal colon and primary colon adenocarcinoma samples from four patients (CRC patients 2-5). Two levels of magnification from a representative field out of three are shown. **f,** Immunohistochemistry for endogenous POU5F1B in TcGT-negative SW480 and HT29 and TcGT-positive LS1034 and HT55 CRC cell lines. A representative picture out of three is shown for each cell line. **g,** Immunohistochemistry (IHC)-score and quantitative PCR (three technical replicates are represented) of LTR66-POU5F1B mRNA from four CRC cell lines (in panel f), CRC patient 1 (in Figure 4b), and CRC patients 2-5 (in panel e). Data in (g) are presented as mean values ± s.d., with single values as circles. Source data are provided as a Source Data file.

Supplementary Figure 5

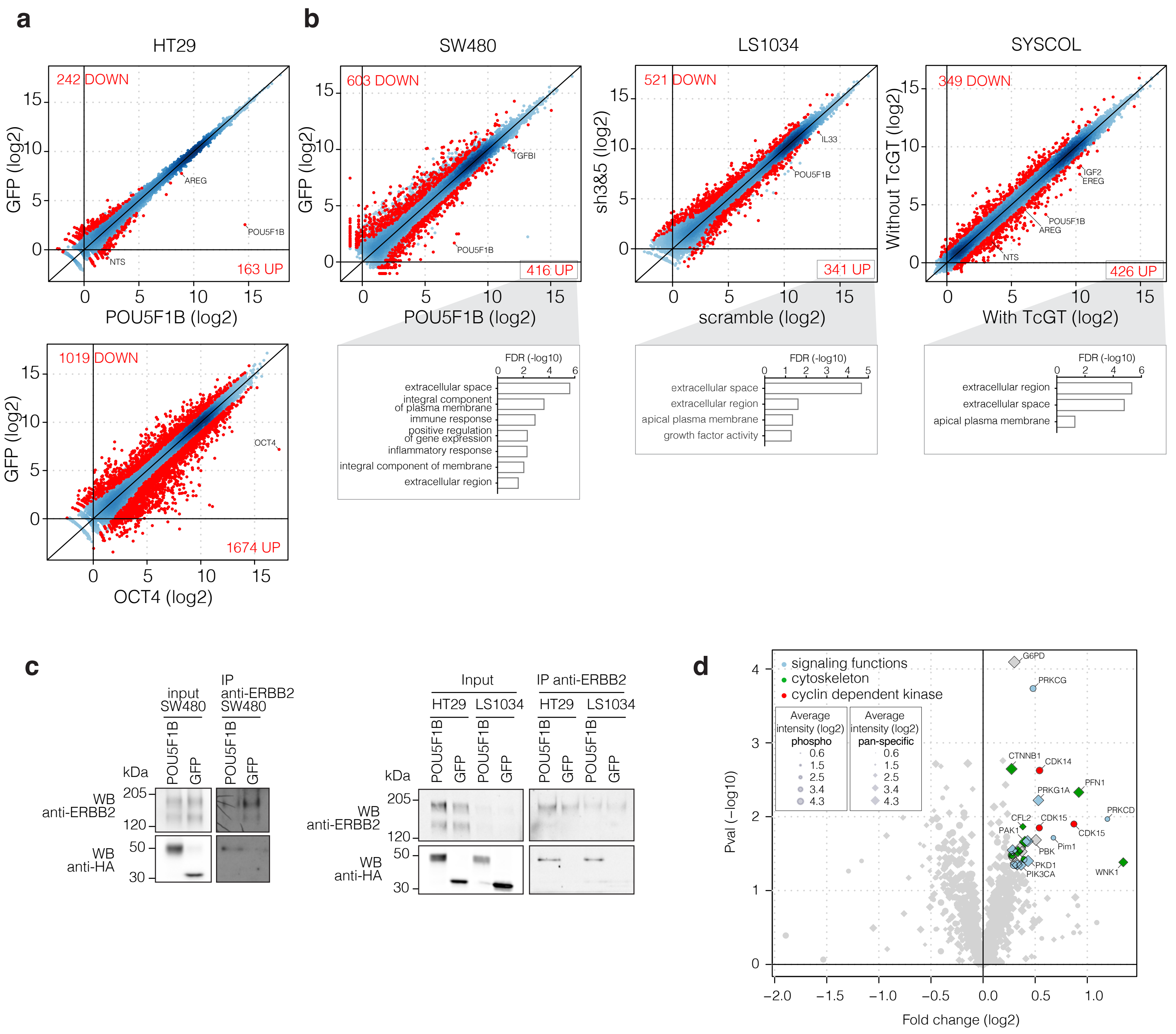

Supplementary Figure 5. Transcriptomic changes upon *OCT4* and *POU5F1B* expression in CRC, confirmation of POU5F1B interaction with ERBB2, and KinexTM array in SW480 cells. a, RNA sequencing analysis of HT29 POU5F1B and GFP overexpressing cells (above) and OCT4 and GFP overexpressing cells (below). **b,** From left to right, RNA sequencing analysis of SW480 POU5F1B vs. GFP overexpressing cells, of sh-scramble vs. shRNA3 & shRNA5 LS1034 cells, and of POU5F1B TcGT expressing vs. non-expressing SYSCOL CRC patients. Upregulated genes were clustered in GO terms using the Functional Annotation Chart from DAVID bioinformatics resources 6.8. Dot plots in **a** and **b** display the log2 average gene expression of three independent replicates from each cell type, red dots correspond to differentially expressed genes (pval < 0.05 by moderated t-test and corrected for multiple testing using the Benjamini-Hochberg's method, and FC>2). **c,** ERBB2 immunoprecipitation in SW480, HT29 and LS1034 GFP-HA- and POU5F1B-HA-overexpressing cells, blotted with anti-HA antibody. A representative experiment out of three is shown. **d,** Volcano plot illustrating relative abundance and phosphorylation of proteins detected with the KinexTM antibody array in total cell extracts of POU5F1B- vs. GFP-overexpressing SW480 cells. Twenty-eight candidates were significantly enriched in POU5F1B-expressing cells (outlined in black), including proteins with signaling functions (blue), related to cytoskeleton (green), and cyclin-dependent kinases (red) (pval < 0.05 by two way Anova test, average spot intensity>1000, average fold change between the two arrays >1.2 and < -1.2). Phospho-proteins are represented as circles and pan-specific detections as diamonds. Source data are provided as a Source Data file.

Supplementary Figure 6

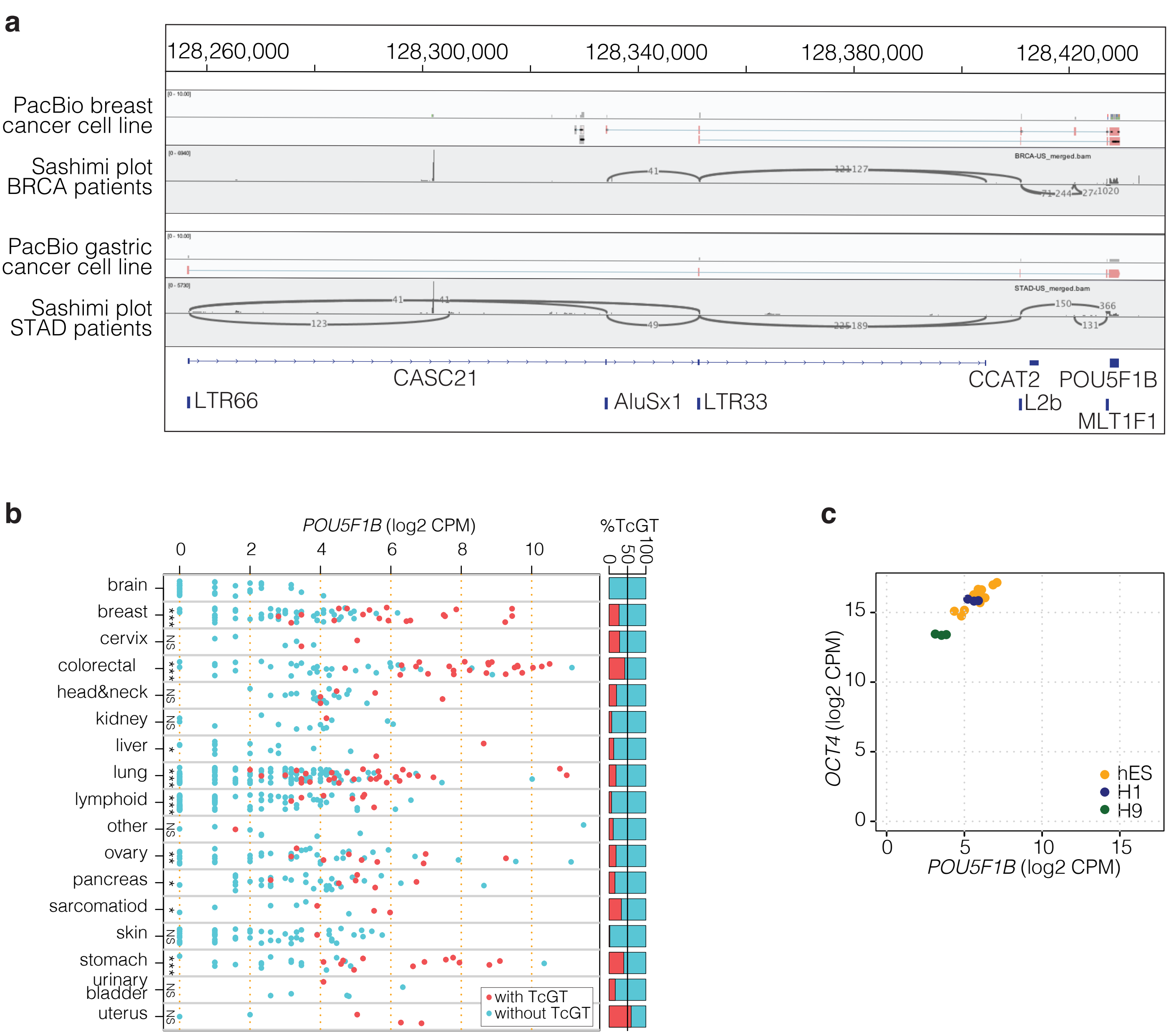

Supplementary Figure 6. Other TEs are promoters of POU5F1B-TcGTs in non-colorectal cancers. POU5F1B is highly expressed in several cancer cell lines and lowly expressed in human embryonic stem cells. a, PacBio and RNA-seq data are displayed in the Integrative Genomics Viewer (IGV). Human genome hg19 chr8 coordinates, coverage and long reads mapped to the reference genome from PacBio, coverage and splice junctions in positive strand from RNAseq (Sashimi plot), annotated genes and TEs are depicted. Data from breast and gastric cancers are shown. **b,** Levels of expression of POU5F1B transcripts in a collection of >600 cancer cell lines (EGAD00001000725)[18]. For each cancer cell type, expression levels in TcGT-containing and TcGT-devoid cell lines were compared with a two-sided t-test. Significance levels are shown as stars (*** pval<0.001, ** pval<0.01, * pval<0.05). **c,** Expression of POU5F1B and OCT4 in human embryonic stem cells. Data from H1 and H9 cell lines, and a collection of clones (hES) are represented[66-67].
