# Supplementary material for: Transposon-activated POU5F1B promotes colorectal cancer growth and metastasis
Source: Nat Commun. 2022 Aug 20;13:4913. doi: 10.1038/s41467-022-32649-7 (PMC9392749; doi:10.1038/s41467-022-32649-7)
Supplement: Supplementary file 3 — Description of Additional Supplementary Files [file 41467_2022_32649_MOESM3_ESM.pdf]

Description of Additional Supplementary Files

File Name: Supplementary Data 1
Description: Ninety-five TcGTs involving 39 genes were detected in more than 20% of tumors and less than 10% of non-tumoral tissue samples.

File Name: Supplementary Data 2
Description: POU5F1B and 60 proteins highly enriched in POU5F1B-specific immuno-precipitates after affinity purification and mass spectrometry (AP-MS) analysis in HT29 and LS1034 transduced cells.

File Name: Supplementary Data 3
Description: Proteins increased (in black) or downregulated (in blue) in POU5F1B-overexpressing SW480 cells.

File Name: Supplementary Data 4
Description: Proteins increased (in black) or downregulated (in blue) in scramble-shRNA transduced LS1034 cells.

File Name: Supplementary Data 5
Description: Proteins increased (in black) or downregulated (in blue) in the supernatant of POU5F1B-overexpressing SW480 cells.

File Name: Supplementary Data 6
Description: Proteins increased (in black) or downregulated (in blue) in the supernatant of scramble-shRNA transduced LS1034 cells.

File Name: Supplementary Data 7
Description: Proteins enriched in POU5F1B-overexpressing SW480 cells (KinexTM array).
