# Supplementary material for: Transposon-activated POU5F1B promotes colorectal cancer growth and metastasis
Source: Nat Commun. 2022 Aug 20;13:4913. doi: 10.1038/s41467-022-32649-7 (PMC9392749; doi:10.1038/s41467-022-32649-7)
Supplement: Supplementary file 10 — Reporting Summary [file 41467_2022_32649_MOESM10_ESM.pdf]

Corresponding author(s): Didier Trono
Laia Simo-Riudalbas

Last updated by author(s): Jul 21, 2022

Reporting Summary

Nature Portfolio wishes to improve the reproducibility of the work that we publish. This form provides structure for consistency and transparency in reporting. For further information on Nature Portfolio policies, see our Editorial Policies and the Editorial Policy Checklist.

Statistics

For all statistical analyses, confirm that the following items are present in the figure legend, table legend, main text, or Methods section.

n/a	Confirmed	
	☒	The exact sample size (*n*) for each experimental group/condition, given as a discrete number and unit of measurement
	☒	A statement on whether measurements were taken from distinct samples or whether the same sample was measured repeatedly
	☒	The statistical test(s) used AND whether they are one- or two-sided<br>*Only common tests should be described solely by name; describe more complex techniques in the Methods section.*
	☒	A description of all covariates tested
	☒	A description of any assumptions or corrections, such as tests of normality and adjustment for multiple comparisons
	☒	A full description of the statistical parameters including central tendency (e.g. means) or other basic estimates (e.g. regression coefficient) AND variation (e.g. standard deviation) or associated estimates of uncertainty (e.g. confidence intervals)
	☒	For null hypothesis testing, the test statistic (e.g. F, t, r) with confidence intervals, effect sizes, degrees of freedom and P value noted<br>*Give P values as exact values whenever suitable.*
☒		For Bayesian analysis, information on the choice of priors and Markov chain Monte Carlo settings
☒		For hierarchical and complex designs, identification of the appropriate level for tests and full reporting of outcomes
	☒	Estimates of effect sizes (e.g. Cohen's *d*, Pearson's *r*), indicating how they were calculated

Our web collection on statistics for biologists contains articles on many of the points above.

Software and code

Policy information about availability of computer code

Data collection	TCGA and GTEx data were downloaded using gdc-client version 1.6.1.<br>Bulk RNA sequencing was performed in the Illumina hiSeq 2000 instrument.<br>qPCR was performed with the Applied Biosystems QuantStudio 6 Flex Real-Time PCR System.<br>Mass Spectrometry data was acquired with Orbitrap Fusion™ Lumos™ mass spectrometer.<br>Absorbance measurements for MTT assays were performed with the Spark Tecan multimode microplate reader.<br>Immunofluorescence pictures were acquired with Zeiss LSM 700 Inverted microscope, using the ZEN 2009 software.<br>Western blot development was done with FUSION FX7 Imaging System.<br>Bioluminiscence imaging was performed on an IVIS Spectrum In Vivo Imaging System (Perkin Elmer) and analyzed with Living Image Software (Perkin Elmer).<br>Pictures from mice tumors and livers were taken with a Nikon DSLR camera.<br>Images from Hematoxylin&Eosin stains were acquired on an Olympus VS120 Whole Slide Scanner, using a 20x objective (UPLSAPO, N.A. 0.75) and a color camera (Pike F505 Color) with an image pixel size of 0.345 microns.
Data analysis	Publicly available computational tools to analyze RNA-seq data, proteomics data, and to build the code for the Transpochimeric Gene Transcript analysis were used as described in the Methods section and they include:

samtools (1.4), bedtools (2.30.0), hisat (2.1.0), subread (1.5.2), stringtie (1.3.4c), MaxQuant (1.6.10.43), DAVID (6.8), R (4.0.2), python (3.6.1), QuPath (0.3.2), R package lsmeans (2.30), lima (3.46.0).
Detailed analysis of micrometastases quantification can be found in the following Zenodo project https://doi.org/10.5281/zenodo.6523649.
ImageJ2 2.3.0 was used for western blot quantification, colony formation assay analysis, and immunofluorescence image processing.
Code for RNAseq processing and Transpochimeric Gene Transcript analysis is published in the Supplementary Code file of this article: https://genome.cshlp.org/content/suppl/2021/08/16/gr.275133.120.DC1.

For manuscripts utilizing custom algorithms or software that are central to the research but not yet described in published literature, software must be made available to editors and reviewers. We strongly encourage code deposition in a community repository (e.g. GitHub). See the Nature Portfolio guidelines for submitting code & software for further information.

Data

Policy information about availability of data

All manuscripts must include a data availability statement. This statement should provide the following information, where applicable:
- Accession codes, unique identifiers, or web links for publicly available datasets
- A description of any restrictions on data availability
- For clinical datasets or third party data, please ensure that the statement adheres to our policy

RNA-sequencing data generated and analyzed during the current study are available in the Gene Expression Omnibus under the accession code GSE182467. The token is: svovicuyvpirpwt.
The mass spectrometry proteomics data have been deposited to the ProteomeXchange Consortium via the PRIDE partner repository with the dataset identifiers PXD028034, PXD028035, and PXD028036.

Project accession: PXD028034
Username: reviewer_pxd028034@ebi.ac.uk
Password: IhjLhykd

Project accession: PXD028035
Username: reviewer_pxd028035@ebi.ac.uk
Password: pKLGhC9l

Project accession: PXD028036
Username: reviewer_pxd028036@ebi.ac.uk
Password: LbdlevJM

Raw RNA sequencing reads were downloaded from NCBI's dbGaP for both, the TCGA and the GTEx datasets.
Raw single cell RNA-seq data was downloaded from the European Genome-phenome Archive (EGAD00001002727).
Raw RNA-seq data from cancer cell lines was downloaded from the EGAD00001000725 repository.
RNA-seq data from eighteen CRC patients was downloaded from GEO (GSE50760).
RNA-seq data from H1 and H9 cell lines, and the collection of hESC clones was downloaded from GEO (GSE60945, GSE83765).
Long-range chromatin interactions in three CRC cell lines were obtained from Jäger, R. et al.
TCGA ATAC-seq peaks were downloaded from https://gdc.cancer.gov/about-data/publications/ATACseq-AWG.
Human genome hg19.

Field-specific reporting

Please select the one below that is the best fit for your research. If you are not sure, read the appropriate sections before making your selection.

[x] Life sciences [ ] Behavioural & social sciences [ ] Ecological, evolutionary & environmental sciences

For a reference copy of the document with all sections, see nature.com/documents/nr-reporting-summary-flat.pdf

Life sciences study design

All studies must disclose on these points even when the disclosure is negative.

Sample size

Analyses of publicly available datasets used all available samples and multiple independent datasets were used to corroborate the reproducibility of the findings.
Sample size in the animal experiments was determined in accordance with power analyses done based on previous experiments, where:
a) to achieve a pval <0.05 it was necessary to have, at least, n=7 animals per group;
b) a certain number of animals may have to be excluded from the experiment due to the complexity of the intervention. The graft rate may vary between 80-100% between animal models;
c) it is necessary for the results to be reproducible.
The number of mice depending on the type of tumor implantation varied as follows:
- For the subcutaneous implantation of cell lines we used n = 7-8 animals per condition.
- For intra-splenic injection of cell lines we used n = 7-9 animals per condition.
- For orthotopic implantation of tumor fragments we used n = 10 animals per condition.

	Sample size calculation was not relevant for any other experiment.
Data exclusions	No data were excluded from the analysis, except for Supplementary Figure 2d, in which one SW620 GFP animal died post surgery.
Replication	All in vitro experiments were reproduced a minimum of 2 to 3 independent times with 3 to 12 technical replicates each, using the same experimental approach. Number of replicates and sample sizes are provided for each of the experiments in figure legends. Findings and conclusions in the manuscript represent those that were consistent across independent experiments. Attempts at replication were successful. In line with the 3R principles (Reduce, Refine, Replace), in vivo experiments were performed once each, albeit ensuring that sample size was appropriate to reach statistical significance.
Randomization	Animal randomization was achieved by having mice injected with different conditions within the same cage. Randomization was not relevant for other experiments described in this study.
Blinding	All in vitro and in vivo experiments were conducted by experimenters blinded to the experimental conditions. When possible, control and test condition were seeded into the same plate to avoid possible technical biases.

Reporting for specific materials, systems and methods

We require information from authors about some types of materials, experimental systems and methods used in many studies. Here, indicate whether each material, system or method listed is relevant to your study. If you are not sure if a list item applies to your research, read the appropriate section before selecting a response.

Materials & experimental systems

n/a	Involved in the study
☐	☒ Antibodies
☐	☒ Eukaryotic cell lines
☒	☐ Palaeontology and archaeology
☐	☒ Animals and other organisms
☒	☐ Human research participants
☒	☐ Clinical data
☒	☐ Dual use research of concern

Methods

n/a	Involved in the study
☒	☐ ChIP-seq
☒	☐ Flow cytometry
☒	☐ MRI-based neuroimaging

Antibodies

Antibodies used	> anti-HA antibody, clone 3F10 Roche 12013819001, 1:1,000, used for western blot, ChIP, immunoprecipitation.<br>> anti-H3K4me3, Cell Signaling 9751, used for ChIP.<br>> anti-H3K27ac, Abcam 4729, used for ChIP.<br>> anti-H3K4me1, Diagenode 037-050 , used for ChIP.<br>> anti-lamin-B1, abcam ab16048, 1:1,000, used for western blot.<br>> rabbit polyclonal custom anti-POU5F1B antibody (1:1,000) ordered from Biotem (https://www.biotem-antibody.com/) and obtained using as immunogen a 16 aa peptide (GDGPWGAEPGWVDPLT), 3 residues of which (underlined) differ between POU5F1B and OCT4. Used for immunohistochemistry.<br>> anti-beta-tubulin, Sigma T4026, 1:1,000, used for western blot.<br>> calnexin, Bethyl A303-696A, 1:2,000 > lamin B1, Abcam ab16048, 1:1,000, used for western blot.<br>> caveolin 1, Santa Cruz sc-894, 1:500, used for western blot.<br>> transferrin receptor, ThermoFisher 13-6800, 1:1,000, used for western blot.<br>> anti-ERBB2 antibody, ThermoFisher BMS120BT, 3ug, used for immunoprecipitation.<br>> anti-ERBB2 antibody, ThermoFisher MA5-13102, 1:100, used for western blot.<br>> HRP-conjugated anti-rabbit, Santa Cruz sc-2004, 1:10,000, secondary antibody used for western blot.<br>> HRP-conjugated anti-mouse, GE Healthcare NA931V, 1:10,000, secondary antibody used for western blot.
Validation	The anti-POU5F1B antibody was extensively validated in the lab upon arrival. OCT4 and POU5F1B positive and negative cells were used for immunohistochemistry validation.<br><br>The other antibodies were used under the manufacturer specifications, being the vast majority of them validated previously in our lab:<br><br>> Anti-HA-Biotin, High Affinity (3F10) is a monoclonal antibody for the highly sensitive detection of HA-tagged recombinant proteins, Fab fragments, conjugated to biotin. The Anti-HA-Biotin, High Affinity antibody (clone 3F10) recognizes the same epitope as clone 12CA5. It is a monoclonal antibody whose high affinity and low working concentrations result in less cross-reactivity than with other antibodies to the HA-epitope. Anti-HA-Biotin, High Affinity (3F10) is a biotin conjugate of this clone which is specifically useful in western blotting, ELISA applications and assays using the universal biotin-streptavidin platform, by allowing specific and highly sensitive detection of HA-tagged proteins.

It has been used in studies described in thousands of papers since its first commecrcialization.
We used it for more than a two hundred ChIPs in Imbeault et al., Nature 2017 543: 550-554.
Other references :

1 Kolodziej, P.A. & Young, R.A. Epitope Tagging and Protein Surveillance. Meth. Enzymol. 194, 508-511.
2 Wilson, I.A., Niman, H.L., Houghten, R.A., Cherenson, A.R., Connolly,M.L. & Lerner, R.A. (1984): The structure of an antigenic determinant in a protein. Cell 37, 767-778.
3 Field, J., Nikawa, J.-I., Broek, D., MacDonald, B., Rodgers, L., Wilson, I.A., Lerner, R.A. & Wigler, M. (1988): Purification of a RAS Responsive Adenylyl Cyclase Complex from Saccharomyces cerevisiae by Use of an Epitope Addition Method. Mol. Cell. Biol. 8, 2159-2165.
4 von Zastrow, M. & Kobilka, B.K. (1992): Ligand-regulated Internalization and Recycling of Human ß2-Adrenergic Receptors between the Plasma Membrane and Endosomes Containing
Transferrin Receptors. J. Biol. Chem. 267, 3530-3538.
5 Emrich, T., Förster, R. & Lipp, M. (1994): Topological Characterization of the Lymphoid-Specific Seven Transmembrane Receptor BLR1 by Epitope-Tagging and High Level Expression. Biochem. Biophys. Res. Com. 197, 214-220.
6 Qian, N.-X., Winitz, S. & Johnson, G.L. (1993): Epitope-tagged Gqa subunits: Expression of GTPase-deficient a subunits persistently stimulates phosphatidylinositol-specific phospholipase C but not mitogen-activated protein kinase activity regulated by the M1 muscarinergic acetylcholin receptor. Proc. Natl. Acad. Sci. 90, 4077-4081.
7 Chen, Y.-T., Holcomb, C. & Moore, H.-P.H (1993): Expression and localization of two low molecular weight GTP-binding proteins, Rab8 and Rab19, by epitope tag. Proc. Natl. Acad. Sci. 90, 6508-6512.
8 Harlow, E. & Lane, D. (1988): Antibodies: A Laboratory Manual, Cold Spring Harbour Laboratory Press, Cold Spring Harbour, N.Y.

> Anti-H3K4me3, Cell Signaling 9751 The manufacturer indicates that antibody specificity was determined by Western blotting. HeLa and NIH/3T3 cell lysates were probed with Tri-Methyl Histone H3 (Lys4) (C42D8) Rabbit mAb (Panel A) or Tri-Methyl Histone H3 (Lys4) Rabbit mAb pre-adsorbed with 1.5 µM of various competitor peptides (Panels B-M). A Western blot is shown where only the tri-methyl histone H3 (Lys4) peptide competed away binding of the antibody. More than 380 citations are listed of published studies making use of this antibody, including for ChIP.

> Anti-H3K27ac, Abcam 4729 This antibody is a rabbit polyclonal to Histone H3 (acetyl K27) - ChIP Grade, raised against a synthetic peptide corresponding to Human Histone H3 aa 1-100 (acetyl K27) conjugated to keyhole limpet haemocyanin. It is suitable for ICC/IF, WB, IHC-P, ChIP, PepArr, and is cited in 1539 publications according to the manufacturer.

> Anti-H3K4me1, Diagenode 037-050 is a polyclonal antibody raised in rabbit against histone H3 containing the monomethylated lysine 4 (H3K4me1), using a KLH-conjugated synthetic peptide. >The Diagenpde website (https://www.diagenode.com/en/p/h3k4me1-polyclonal-antibody-classic-50-ug-18-ul) provides illustrations of the specificity of the antibody via ChIP PCR in HeLa cells, with primers for a region surrounding the ACTB and GAS2L1 genes, used as positive controls, and for the promoters of the GAPDH and EIF4A2 genes, used as negative controls.

> Anti-lamin-B1, abcam ab16048 is a rabbit polyclonal raised against a synthetic peptide. The manufacturer states that performance for WB has been assessed in: HeLa, PC12 and NIH/3T3 whole cell lysate, wild type HAP1 whole cell lysate, wild type HAP1 nuclear lysate, human pancreatic cell line whole cell lysate. It has been referenced in 858 publications according to Abcam.

> Anti-beta-tubulin, Sigma T4026 is a mouse monoclonal referenced in 932 publications according to the supplier, recognizing beta-tubulin from wheat, mouse, hamster, sea urchin, frog, plant, bovine, rat, rabbit, moth, chicken and human, and recommended for WB and IF.

> Anti-calnexin, Bethyl A303-696A is a rabbit polyclonal recommended for IHC, IP and WB, recognizing protein from mouse and human. Previously used by our lab and cited in:
1. Hydrostatic pressure promotes migration and filamin-A activation in fibroblasts with increased p38 phosphorylation and TGF-β production. Kao, Chen, Wang et al. Biochem Biophys Res Commun (2021) 568, 15-22 DOI: 10.1016/j.bbrc.2021.06.055.
2. Transposable elements and their KZFP controllers are drivers of transcriptional innovation in the developing human brain. Playfoot et al. Genome Res. 2021 Sep;31(9):1531-1545. doi: 10.1101/gr.275133.120. Epub 2021 Aug 16. PMID: 34400477.

> Anti-caveolin 1, Santa Cruz sc-894 is a rabbit polyclonal raised against a peptide derived from the N-terminus of the human protein. It is referenced in 312 publications according to the manufacturer.

> Anti-transferrin receptor, ThermoFisher 13-6800 is a monoclonal antibody, the specificity of which was verified by using knockdown cell lines. It is referenced in 480 publications according to the supplier.

> Anti-ERBB2 antibody, ThermoFisher BMS120BT is a mouse monoclonal antibody raised against the human protein. Recommended for FACS and IP. the epitope is located in the extracellular domain, no cross reactivity with EGF-R has been detected by immunoprecipitation. Cited in several hundred publications.

> Anti-ERBB2 antibody, ThermoFisher MA5-13102 is a mouse monoclonal antibody recommended for Western blot analysis by the supplier. In an illustration of performance on Thermofisher website (https://www.thermofisher.com/antibody/product/ErbB2-HER-2-Antibody-clone-e2-4001-Monoclonal/MA5-13102), a 185 kDa band corresponding to ErbB2 was observed in SK-BR-3 but not in MDA-MB-231, which is a negative control for ErbB2. According to supplier, cited in 23 references for WB alone.

> HRP-conjugated anti-rabbit Santa Cruz sc-2004 is a goat polyclonal antibody conjugated to horseradish peroxidase recommended as secondary antibody for WB, and used in hundreds of publications.

> HRP-conjugated anti-mouse, GE Healthcare NA931V is a sheep anti-mouse antibody conjugated to horseradish peroxidase recommended as secondary antibody for Western blot, with 104 references cited by the supplier.

Eukaryotic cell lines

Policy information about cell lines

Cell line source(s)	> LS1034 and NCI-H508 cell lines were obtained from the American Type Culture Collection (ATCC) and maintained in RPMI 1640 medium (Gibco) supplemented with 10% FCS (Bioconcept 2-01F36-I);<br>> LS174T (ATCC) and HT55 (Sigma) cells were cultured in EMEM supplemented with 10% FBS, HT55 being complemented with 2 mM glutamine (Gibco) and 1% of non-essential aminoacids (Sigma);<br>> SW480 and SW620 (ATCC) were cultured in L15 medium (Sigma);<br>> HT29 (ATCC) in McCoy's 5A (Thermo Fisher);<br>> LoVo (ATCC) in Ham's F12K (ThermoFisher);<br>> DLD1 (ATCC) in RPMI 1640 medium (Gibco) supplemented with 10% FCS (Bioconcept 2-01F36-I);<br>> HCT116 (ATCC) in DMEM (Gibco), being all supplemented with 10% FCS.<br>> For lentiviral vector production, 293T cells (ATCC) were cultured in DMEM supplemented with 10% FBS with 100 IU ml-1 penicillin, 100 ug ml-1 streptomycin, and 26 µg ml-1 glutamine (Corning 30-009-CI) at 37°C in a humidified atmosphere of 5% CO_2.
Authentication	Since they were purchased from official cell lines repositories, none of the cell lines used were authenticated.
Mycoplasma contamination	All cell lines were tested negative for mycoplasma contamination.
Commonly misidentified lines (See ICLAC register)	No commonly misidentified cell lines were used in the study.

Animals and other organisms

Policy information about studies involving animals; ARRIVE guidelines recommended for reporting animal research

Laboratory animals	Male NOD scid gamma (NSG) immunodeficient mice 7-9 weeks old were used in this study. Animals were maintained under standard animal housing conditions in a normal 12-h light–dark cycle with ad libitum access to food and water.
Wild animals	No wild animals were used in the study.
Field-collected samples	No field-collected samples were used in the study.
Ethics oversight	All animal experiments were performed within the EPFL animal facility, in accordance with the Swiss Federal Veterinary Office guidelines and as authorized by the Cantonal Veterinary Office (animal license VD3381).

Note that full information on the approval of the study protocol must also be provided in the manuscript.
